# Druggable genomic landscapes of high-grade gliomas

**DOI:** 10.3389/fmed.2023.1254955

**Published:** 2023-12-08

**Authors:** Paola Ghanem, Maria Fatteh, David Olayinka Kamson, Archana Balan, Michael Chang, Jessica Tao, Jaishri Blakeley, The Johns Hopkins Molecular Tumor Board Investigators, Christine Pratilas, Taxiarchis Botsis, Rena Xian, Chris Gocke, Tseh Ming-Lin, Eitan Halper-Stromberg, Ying Zou, Kent Hardart, Jonathan Spiker, Kory Kreimeyer, Ting He, Katie Fiallos, Dana Petry, Kala Visvanathan, Antonio Wolff, Cesar Santa-Maria, Raquel Nunez, Christian Meyer, John Laterra, Vered Stearns, Karen Smith, Deborah Armstrong, Rachel Karchin, Katerina Karaindrou, Lily Zandi, Marta Majcherska, Faith Too, Monique Makell, Jennifer Lehman, Timsy Wanchoo, Jaime Wehr, Michael Conroy, Selina Shiqing Teh., Jenna Canzoniero, Stuart A. Grossman, Kristen Marrone, Karisa C. Schreck, Valsamo Anagnostou

**Affiliations:** ^1^Department of Oncology, The Sidney Kimmel Comprehensive Cancer Center, Johns Hopkins University School of Medicine, Baltimore, MD, United States; ^2^The Johns Hopkins Molecular Tumor Board, Johns Hopkins University School of Medicine, Baltimore, MD, United States; ^3^Department of Neurology, Johns Hopkins University School of Medicine, Baltimore, MD, United States

**Keywords:** genomic landscape, glioblastoma, glioma, actionable mutation, precision oncology, molecular tumor board, targeted therapies

## Abstract

**Background:**

Despite the putatively targetable genomic landscape of high-grade gliomas, the long-term survival benefit of genomically-tailored targeted therapies remains discouraging.

**Methods:**

Using glioblastoma (GBM) as a representative example of high-grade gliomas, we evaluated the clonal architecture and distribution of hotspot mutations in 388 GBMs from the Cancer Genome Atlas (TCGA). Mutations were matched with 54 targeted therapies, followed by a comprehensive evaluation of drug biochemical properties in reference to the drug’s clinical efficacy in high-grade gliomas. We then assessed clinical outcomes of a cohort of patients with high-grade gliomas with targetable mutations reviewed at the Johns Hopkins Molecular Tumor Board (JH MTB; *n* = 50).

**Results:**

Among 1,156 sequence alterations evaluated, 28.6% represented hotspots. While the frequency of hotspot mutations in GBM was comparable to cancer types with actionable hotspot alterations, GBMs harbored a higher fraction of subclonal mutations that affected hotspots (7.0%), compared to breast cancer (4.9%), lung cancer (4.4%), and melanoma (1.4%). In investigating the biochemical features of targeted therapies paired with recurring alterations, we identified a trend toward higher lipid solubility and lower IC_50_ in GBM cell lines among drugs with clinical efficacy. The drugs’ half-life, molecular weight, surface area and binding to efflux transporters were not associated with clinical efficacy. Among the JH MTB cohort of patients with *IDH1* wild-type high-grade gliomas who received targeted therapies, trametinib monotherapy or in combination with dabrafenib conferred radiographic partial response in 75% of patients harboring *BRAF* or *NF1* actionable mutations. Cabozantinib conferred radiographic partial response in two patients harboring a *MET* and a *PDGFRA/KDR* amplification. Patients with *IDH1* wild-type gliomas that harbored actionable alterations who received genotype-matched targeted therapy had longer progression-free (PFS) and overall survival (OS; 7.37 and 14.72 respectively) than patients whose actionable alterations were not targeted (2.83 and 4.2 months respectively).

**Conclusion:**

While multiple host, tumor and drug-related features may limit the delivery and efficacy of targeted therapies for patients with high-grade gliomas, genotype-matched targeted therapies confer favorable clinical outcomes. Further studies are needed to generate more data on the impact of biochemical features of targeted therapies on their clinical efficacy for high-grade gliomas.

## 1 Introduction

Glioblastoma (GBM) is the most aggressive primary brain cancer and represents 48% of malignant brain tumors, with a dismal 5-year overall survival (OS) rate of 5.5% (1). To date, resection followed by radiotherapy in combination with temozolomide (TMZ) constitutes the standard of care for newly diagnosed GBM (2). Recurrence remains virtually inevitable even if the chemotherapy and radiation are preceded by gross total resection of the contrast enhancing lesion (3). This phenomenon may be attributed to the fact that diffuse gliomas extend far beyond the contrast enhancing areas, or even the radiographically abnormal volume (4–6). Failure of systemic therapies to adequately concentrate into areas of the brain with an intact blood brain barrier is likely one of the major factors limiting the success rate of current treatments (7). The molecular characterization by *IDH*-mutation subtype along with the MGMT promoter methylation profile is increasingly important for accurate prognostication and treatment administration (8). With the increased use of next-generation sequencing, analyses of the genomic background of GBM have identified multiple putatively actionable mutations in driver genes, including in *EGFR*, *CDK4*, *PDGFRA, CDKN2A/2B, BRAF*, and *PTEN* (9). Several core signaling pathways are dysregulated in GBM, including the RTK/Ras/PI3K pathway, and the TP53 and RB1 regulated cell cycle progression programs (10). Despite the expanding landscape of putatively actionable alterations of GBM, the clinical success rate of molecularly driven therapies that target these alterations remains limited with only one FDA-approved targeted therapy for patients with gliomas (11). To date, several host, cancer, and drug-related factors limit the efficacy of such therapies among which are GBM clonal heterogeneity, differential glioma stem cell drug sensitivity, drug trafficking across the blood brain barrier (BBB), and drugs biochemical properties. More specifically, the genomic heterogeneity of GBM, reflected in sub-clonal cancer cell populations carrying distinct sets of genomic alterations may confer bypass resistance mechanisms and therefore contribute to a fitness advantage of GBM in the context of single agent targeted therapy and treatment failure (12). Furthermore, there are limited comprehensive analyses to support potential correlations between the biochemical features and clinical efficacy of targeted therapies.

Here, we assessed the clonal architecture of GBM and determined the frequency of clonal and subclonal actionable alterations. We next evaluated the biochemical properties of molecularly tailored therapies targeting these alterations with respect to their efficacy in clinical trials. Lastly, we used this approach to interpret clinical outcomes of patients with high-grade gliomas who received targeted therapies tailored to the tumor’s genetic profile after review at the Johns Hopkins Molecular Tumor Board.

## 2 Materials and methods

### 2.1 Characterization of GBM genomic alterations

We evaluated 388 GBMs from The Cancer Genome Atlas (TCGA). Whole exome sequencing (WES)-derived somatic mutation calls from the TCGA Atlas MC3 project were retrieved from the NCI Genomic Data Commons^1^ (13). Recurring sequence mutations (hotspots) were defined as sequence alterations with ≥10 entries in any tumor type in COSMIC (14). We subsequently used OpenCRAVAT to annotate missense mutations and determine putative pathogenic variants (15, 16). Somatic copy number profiles, tumor purity and ploidy estimates and allele-specific copy number states (17) were derived from the pancancer atlas,^2^ focal copy number alterations were subsequently determined as previously described (18). Mutation cellular fraction (CF), that is the fraction of cancer cells harboring a specific mutation, was estimated for each mutation using mutant allele fraction along with estimates of tumor purity and ploidy as previously described (18). To account for any over estimation of tumor purity in computing CF, we assessed cellular fractions after reducing tumor purity by 10 and 20% of the original estimate. The following criteria was then used to assign clonality: (1) mutations with CF < 0.75 for both the reduced purity estimates were assigned as subclonal; (2) mutations with CF ≥ 0.75 for both the reduced purity estimates were assigned as clonal; (3) mutations which did not fulfill either of these criteria were dropped from clonality assessment.

We restricted our analyses to genes mutated in >1% of samples and involved in cancer signaling pathways/cancer hallmarks, to ensure that rare driver alterations are captured. These mutations encompassed 1,651 sequence and structural alterations across 367 samples. It included a total of 1,156 sequence alterations that were evaluated for hotspots and clonality was assessed for 904 sequence alterations. A summary of the methodology is provided in the Supplementary Figure S1. Frequently deregulated pathways and hallmarks including cell cycle progression, PI3K/AKT pathway, RAS/RAF pathway, tyrosine kinase signaling, DNA damage repair, chromatin regulation, NOTCH signaling and Sonic Hedgehog signaling were considered (Supplementary Figure S2). To further understand the genomic diversity of GBM as compared to other cancer types, we assessed WES-derived somatic mutation calls from the TCGA Atlas MC3 project (13) across different cancer types (Supplementary Figure S3) and computed the fraction of hotspot alterations alongside their cellular fractions, restricting our analysis to the commonly deregulated pathways observed in GBM.

### 2.2 Biochemical features of targeted therapies

We extracted biochemical data for drugs targeting hotspot mutations occurring in GBM and restricted our analysis to genes with a mutation rate of ≥1% based on the TCGA data. Biochemical properties were collected for 54 drugs, grouped into the following categories: BRAF, PARP, CDK4/6, EGFR, PIK3CA, PDGFRA, EZH2, IDH1, KIT, KDR, MET, MEK, ERK, AKT, FGFR, mTOR, SHP2, and ROS1 inhibitors. The classes of drugs included both direct and indirect inhibitors of putatively actionable hotspot mutations in GBM. Drugs included were FDA-approved agents against actionable mutations and/or agents tested in phase 1–3 trials in primary brain tumors. Molecular weight (MW), surface area (SA), charge, lipid solubility [measured by the XLogP3-AA parameter which takes into account the elements of the chemical structure and number of hydrogen bonds among other factors (17)], reversibility and half-life of each drug were extracted from PubChem (18). SA, MW and lipid solubility were further classified into high, moderate and low categories based on quartiles (high: >75th percentile, moderate: 50th–75th percentile, and low <50th percentile; Supplementary Table S1). Data on drug IC_50_ in cell-free based assays (nM) and in GBM cell lines (μM; A172, T98G, LN-18, LN-229, U138, U87, U18, LN-405, SF188) were extracted from selleckchem.com (Supplementary Table S2). For drugs with multiple GBM-specific IC_50_, the median IC_50_ was used for statistical analyses.

### 2.3 Drug distribution in brain and blood compartments

To determine the presence of efflux pumps, brain-to-blood ratio as well as the preclinical and clinical maximal plasma and brain (tumor/non-tumor/CSF) concentrations, we generated a PubMed query with the following Mesh terms used in the search strategy: (“Brain neoplasms” OR “glioma” OR “glioblastoma”) AND (“blood brain barrier” OR “brain/metabolism” OR “brain/drug effects” OR “tissue distribution” OR “blood brain barrier/metabolism” OR “blood brain barrier/drug effects” OR “efflux pumps”), along with each of the targeted agents investigated in this study (Supplementary Table S3). A total of 398 articles were retrieved. The studies’ objectives and results were first evaluated by reviewing the abstracts. Studies that fulfilled the aim of assessing the pharmacodynamic and pharmacokinetic properties of those targeted agents were then fully reviewed (*n* = 116). Preclinical (animal model-derived) and clinical (human-derived) data on the maximal plasma and brain concentrations were extracted from each study and are summarized in Supplementary Table S3. The presence of efflux pumps was extracted from preclinical studies (Supplementary Table S3).

### 2.4 Assessment of clinical efficacy of targeted therapies

To acquire published studies supporting the clinical efficacy of targeted therapies in primary brain tumors along with their maximal tolerated dose, we generated a PubMed query as follows: (“Brain neoplasms” OR “glioma” OR “glioblastoma”) AND “molecular targeted therapy” OR “therapeutics” OR “glioblastoma/drug therapy”) AND (“case study” OR “case report” OR “case series” OR “trial” OR “phase” OR “clinical trial, phase I” OR “clinical trial, phase II” OR “maximum tolerated dose,” along with each of the targeted agents investigated in this study (Supplementary Table S3). We limited our search to articles in English and identified additional studies by manual review of citations included in eligible articles. A total of 350 articles were retrieved. The highest available evidence of clinical efficacy was evaluated for each drug and included 75 published articles in our final review. We determined the clinical efficacy of each targeted drug based on their reported clinical efficacy in clinical trials and case reports, employing a scoring system as follows: Clinical efficacy established by phase II-III clinical trials was considered strong evidence (scored as +2) while efficacy established by phase I trials and case reports was considered weaker evidence (scored as +1). Lack of efficacy established in phase II-III trials was considered strong evidence (scored as −2) while lack of efficacy in phase I trials and case reports was considered weaker evidence (scored as −1). Drugs with no available studies supporting their clinical efficacy were given a score of 0. These assessments were performed by two independent neuro-oncologists. Out of 54 drugs assessed, 44 targeted therapies were evaluated in phase I/II clinical trials and case reports and were further included in analyses (Supplementary Table S3).

### 2.5 Primary brain tumors reviewed at the Johns Hopkins molecular tumor board

We extracted clinical data for 63 individuals with primary brain tumors tested by next-generation sequencing that were reviewed at the Johns Hopkins Molecular Tumor Board between June 1, 2014 and April 21, 2023 under an institutionally approved protocol (19). Excluding low-grade gliomas, we limited our analyses to 50 patients with oligodendroglioma (*n* = 1), high-grade astrocytoma (*n* = 15) and glioblastoma (*n* = 34). The average age at diagnosis was 43.0 years ±17.3 years (mean ± standard deviation) with 22 (44.0%) females, 39 (78.0%) white, 7 (14.0%) African American and 2 (4.0%) Asian (Supplementary Table S4). Next-generation sequencing (NGS) data for all 50 high-grade primary brain tumors were retrieved; 44 tumor-only targeted NGS employing 340–592 gene panels (JHU Solid Tumor Panel, FoundationOne CDx, CARIS MI profile) and 6 matched tumor/normal targeted NGS (Cancer Select) covering 88–125 genes. An overview of the sequence and structural alterations detected is shown in the Supplementary Table S5. Hotspot mutations were determined by a COSMIC count of ≥10 occurrences and variant annotation was performed using OpenCRAVAT (15). To study *bona fide* oncogenic alterations and to annotate alterations without robust biochemical characterization of functional consequence, we restricted our analyses to missense alterations with a high driver potential (CHASMplus score ≥ 0.75) as well as putatively loss-of-function truncating mutations (nonsense, frameshift and affecting splice donor/acceptor sites). Non-hotspot alterations with a CHASMplus score < 0.75 (20) were classified as variants of uncertain significance, while non-hotspot variants with a CHASMplus score ≥ 0.75 (20) were classified as oncogenic/likely oncogenic variants (Supplementary Table S5). Putatively oncogenic alterations were identified in all 50 tumors. Eighteen of the 50 patients received targeted therapies paired with targetable alterations detected in the tumor specimens analyzed (Supplementary Table S4). Radiographic response was assessed based on routine local radiology review as part of standard clinical care. Radiographic partial response (PR) was noted in 53% of patients (*n* = 8), three patients (20%) had stable disease (SD) and progressive disease (PD) was noted in four (27%) patients. Radiographic response could not be assessed in 3 patients; two patients were lost to follow up and one did not have any radiographic assessments after treatment initiation in our records (Supplementary Table S4). Overall survival (OS) and progression-free survival (PFS) were computed as time from treatment initiation to death (or last clinical follow up if patient is still alive) and progression, respectively.

### 2.6 Statistical analyses

The Kruskal-Wallis non-parametric test was utilized to evaluate differences in continuous biochemical features (molecular weight, lipid solubility, surface area, brain to blood ratio, half-life) for targeted agents with differential clinical efficacy (Supplementary Table S6). Fisher’s exact test was performed to evaluate the association of categorical features with clinical efficacy; non-parametric tests were employed as these do not assume a normal distribution of the data analyzed. All *p*-values were based on two-sided testing and differences were considered significant at *p-*value ≤ 0.05. Statistical analyses were conducted using the IBM SPSS software, version 27.

## 3 Results

### 3.1 Actionable mutation landscape of glioblastoma

**Figure 1:**
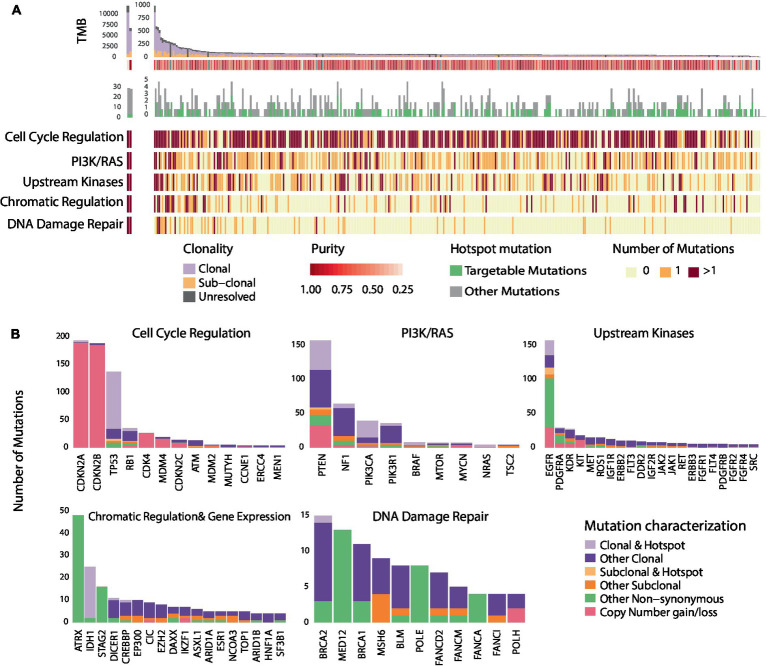
Genomic heterogeneity of glioblastomas and distribution of recurring mutations within cancer hallmarks and gene pathways. **(A)** Illustrates the genomic landscape and clonal composition of the 329 GBMs in the TCGA cohort with clonality assessments. 65.9% of tumors harbored a hotspot sequence alteration, and 46.2% harbored at least one targetable alteration depicted in green. The number of nonsynonymous mutations within each of the five most altered signaling pathways is illustrated; red represents two or more mutations occurring within signaling pathways/cancer hallmarks, orange represents the occurrence of one mutation, and light yellow represents no mutation. Genomic alterations in GBM tumors frequently occur in genes within core signaling pathways and cancer hallmarks. **(B)** Further illustrates the hotspot and sub-clonal genomic alterations within the five altered core signaling pathways. The bar plot represents the number of mutations observed in all the GBMs (*n* = 388) that we evaluated from the TCGA. A sizable fraction of hotspot sequence alterations (28.6%) is observed in GBM. Among alterations with clonality assessments, 84.7% were clonal mutations. The distribution of hotspot clonal mutations in GBM within each gene is represented in light purple, sub-clonal hotspot mutations are shown in light orange, sub-clonal non-hotspot mutations are illustrated in dark orange and clonal non-hotspot mutations are illustrated in dark purple. All remaining non-synonymous alterations are represented in green.

As described in previously reported TCGA GBM studies (10), a number of recurring sequence and structural alterations were identified (Figure 1 and Supplementary Figure S2). Among all sequence alterations evaluated (*n* = 1,156), a sizable fraction involved hotspot mutations (28.6%; Figure 1B), with 56.5% of hotspot mutations comprising targetable alterations (Methods) These encompassed genomics alterations in *EGFR* (5.6%), *PTEN* (4.3%), *PIK3CA* (2.2%), *IDH1* (2.1%), *NF1* (0.7%), and *BRAF* (0.4%; Figure 1B and Supplementary Table S7). The complete targetable genomic landscape of GBM is summarized in the Supplementary Table S7. Focusing on sequence alterations, out of 329 GBMs, 65.9% of tumors harbored a hotspot mutation (Supplementary Table S8). In addition, a sizable fraction of GBMs (46.2%) harbored at least one targetable mutation which included *EGFR* mutations in 17.3%, *PTEN* in 14.9%, *PIK3CA* in 7.3%, *BRAF* in 1.5%, *mTOR* in 0.6%, *BRCA2* in 0.3%, *NF1* in 2.1%, *KDR* in 0.3% and *PDGFRA* in 0.3% of tumors (Supplementary Table S8). In comparing the frequency of hotspot genomic alterations in GBM to that of tumors historically sensitive to molecularly directed therapies targeting these alterations, we identified a comparable rate of hotpot mutation occurring in GBM (28.6%) to that of breast cancer (40.2%), ovarian cancer (38.9%), colon cancer (30.9%) and lung adenocarcinoma (28.2%; Supplementary Figure S3 and Supplementary Table S9).

As intra-tumoral clonal heterogeneity may represent a dominant intrinsic mechanism mediating resistance to targeted therapies, we next examined the genomic heterogeneity of GBM by evaluating the clonal architecture within each tumor (Supplementary Figure S2 and Supplementary Table S10). We found that out of 904 genomic sequence alterations with clonality assessments (Methods), 84.7% of mutations were clonal (Figure 1B and Supplementary Table S10). The fraction of sub-clonal sequence alterations varied by gene; 30% of *PDGFRA*, 28.6% of *EGFR*, 16.6% of *ATM*, 13.0% of *NF1*, 10.1% of *PTEN*, 8.6% of *PIK3CA*, and 5.5% of *TP53* alterations were found to be sub-clonal. Notably, only 2.1% of sequence alterations were hotspot sub-clonal mutations. Among all hotspot sequence alterations identified in GBM, 7.0% were sub-clonal and varied by gene: 32.1% of *EGFR*, 6.5% of *PTEN* and 3.7% of *TP53* hotspot mutations were sub-clonal (Figure 1B and Supplementary Table S10). Out of all recurring sequence mutations, 8.6% were targetable sub-clonal mutations. These findings support the notion that clonal heterogeneity of GBM may affect the clinical efficacy of drugs and potentially drive mixed responses or point to activation of bypass resistance mechanisms. To interpret these findings in the context of different cancer types, we determined the frequency of sub-clonal mutations affecting hotspots in additional tumor types. The frequencies of sub-clonal recurring mutations out of all hotspot mutations, were significantly lower in bladder cancer (5.0%), breast cancer (4.9%), lung cancer (4.4%), head and neck cancer (3.7%), and melanoma (1.4%; Supplementary Figure S3).

**Figure 2:**
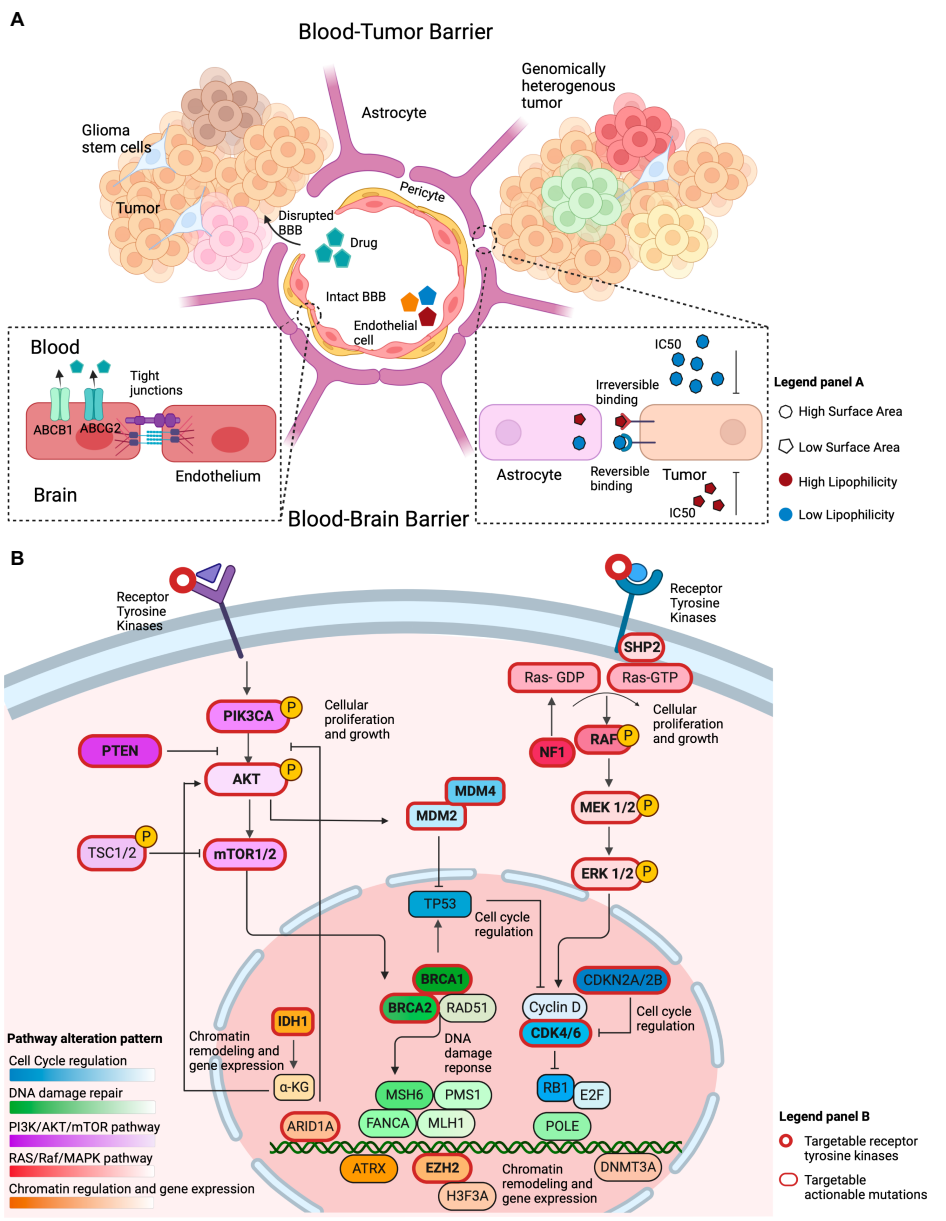
Overview of factors that conceptually affect the efficacy of genotype-matched targeted therapies in brain tumors. **(A)** Illustration of several features that may limit the accumulation of targeted therapies in the brain and their clinical efficacy. The integrity of the blood-brain barrier (BBB) may be compromised in primary brain tumors, allowing drugs to cross into the tumor irrespective of their biochemical properties. The BBB represents the intact barrier composed of endothelial cells, pericytes, and astrocytes that is present in non-enhancing brain regions. The tight junctions at the blood–brain barrier and efflux transporters such as (ABCB1 and ABCG2) limit the ability of drugs to permeate an intact blood–brain barrier. Other biochemical features, such as the drugs’ surface area, lipophilicity, and reversible binding, can also affect the ability of those drugs to concentrate in the brain. Drugs with a small surface area, elevated lipid solubility, and irreversible binding require a lower IC_50_ to adequately inhibit their target on tumor cells. The opposite holds true for drugs with a large surface area, low lipid solubility, and reversible binding. **(B)** Illustration of the most commonly altered signaling pathways observed in GBM, a representative example of high-grade glioma, based on the TCGA data. The PI3K/AKT/mTOR and RAS/Raf/MAPK pathways are frequently altered and conceptually represent a viable therapeutic target. Alterations within cell cycle regulating genes, such as, in *MDM2*, *MDM4*, *CKDN2A/B*, and *CDK4/6* are actionable. Chromatin regulation can be targeted through *IDH1*, *ARID1A*, and *EZH2* targeted therapies. Alterations in DNA damage repair are exemplified by targetable *BRCA1/2* mutations.

We next considered convergence of mutations in genes within signaling pathways and cancer hallmarks that can be targeted by molecularly driven therapies (Figure 2B and Supplementary Figure S2). Alterations in genes involved in cell cycle regulation mediated by *CDKN2A/2B* were identified in 78.1% of GBM tumor samples. Sixty one percent of tumors harbored alterations in genes in the PI3K/Ras signaling pathway, while mutations in tyrosine kinases were identified in 48.9% of cases. Interestingly, out of 388 GBMs, 43 tumors harbored alterations in both the cell cycle regulation pathway and upstream kinases and 42 tumors harbored alterations in the cell cycle regulation and PI3K/Ras pathway. On the contrary, only 10 tumors harbored mutations in PI3K/Ras pathway and upstream kinases. Alterations in genes involved in chromatin regulation and DNA damage repair were identified in 26.0 and 13.7%, respectively, (Figure 1). Interestingly, only a few tumors (*n* < 8) with alterations in the DNA Damage Repair deficiency pathway harbored mutations in other signaling pathways. Less frequently altered pathways included the NOTCH, Hedgehog signaling pathways, and the Wnt signaling pathway (Supplementary Figure S2). Overall, these findings point toward common therapeutic vulnerabilities in GBM that could be leveraged in the clinical setting.

### 3.2 Biochemical properties of molecularly-directed therapies

While comprehensive genomic analyses have revealed multiple actionable sequence and structural alterations in GBM that conceptually imply therapeutic vulnerabilities (10), the clinical efficacy of targeted therapies in this cancer type has been limited. Host, tumor, and drug-related factors summarized in Figure 2 influence the effect of targeted therapies in patients with GBM. To better understand the features of targeted therapies for patients with primary brain tumors that may contribute to their clinical efficacy, we assessed the biochemical properties of drugs directed at frequently occurring mutations. To this end, we hypothesized that a high lipid solubility and a low surface area (SA) are two features that could affect the efficacy of targeted therapies against GBM, as a strong lipophilicity potentially indicates the ability of drugs to penetrate the BBB (21), while the SA could affect the ability of the drug to permeate the brain cell membrane (22). In addition, the presence of efflux transporters at the BBB could offset the efficacy of drugs with such biochemical features. Consistent with our hypothesis, a trend toward a higher lipid solubility was observed for drugs with a proven clinical efficacy in phase 2 trials compared to the lipid solubility of drugs that lacked clinical efficacy (Kruskal Wallis *p*-value: 0.17; Supplementary Table S6). We then postulated that the drug’s half-life would affect its efficacy. Interestingly, there was no statistical difference between the drugs’ half-life and the drug’s clinical efficacy in phase 1 or phase 2 trials compared to drugs that lacked efficacy (Supplementary Table S6). Along with the aforementioned biochemical properties, we hypothesized that the drug’s half-maximal inhibitory concentration in GBM cell lines (IC_50_) is an additional biological factor that is potentially related to the efficacy of targeted therapies to accumulate in non-enhancing brain regions. Targeted therapies that demonstrated clinical efficacy in phase 2 trials compared to drugs that lacked clinical efficacy, showed a trend toward lower IC_50_ in GBM-specific cell lines (mean rank 8.80 vs. 11.64 μM, Kruskal Wallis *p*-value: 0.26; Supplementary Table S6).

**Figure 3:**
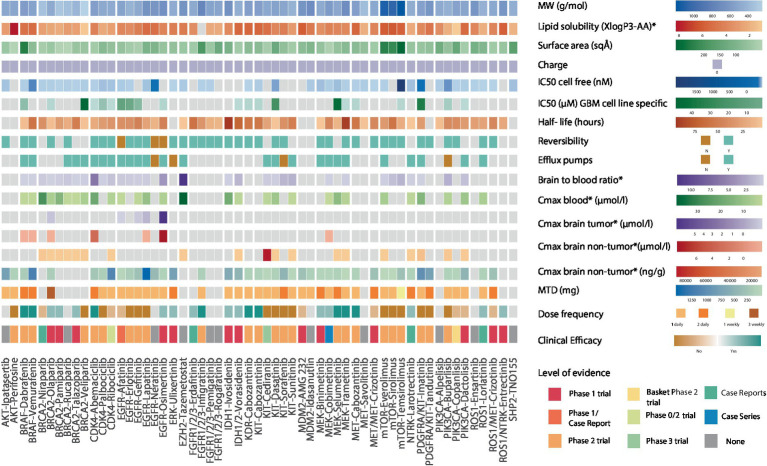
Heatmap showing the biochemical features of 54 genotype-targeted therapies. This figure illustrates the different biochemical features of 54 targeted therapies against the most commonly occurring genomic alterations (≥1%) in GBM, among which the molecular weight, surface area, lipid solubility, charge, half-maximal inhibitory concentration, drugs’ reversible binding to their target, their half-life, and their binding to efflux transporters at the blood brain barrier. The brain-to-blood ratio and the maximal plasma and brain (tumor/non-tumor) concentrations obtained from preclinical studies as well as maximum tolerated dose (MTD), dose frequency and clinical efficacy of drugs are also displayed here. For clinical efficacy, light colors reflect weak evidence from phase 1 trials and case reports/series whereas darker shades of color reflect strong evidence from phase 2 trials. The highest level of evidence for each targeted therapy is also provided in the heatmap. The CDK4/6 inhibitor abemaciclib, the receptor tyrosine kinase (RTK) inhibitor cabozantanib, the BRAF inhibitors dabrafenib and vemurafenib, the FGFR inhibitor infigratinib and the MEK inhibitors selumetinib and trametinib have strong evidence suggesting clinical efficacy. The PARP inhibitors talazoparib, olaparib, and pamiparib, the EGFR inhibitor osimertinib, the ERK inhibitor ulixertinib, the FGFR inhibitor erdafitinib, the IDH inhibitors ivosidenib and vorasidenib, the MEK inhibitors binimetinib and cobimetinib, the NTRK inhibitor larotrectinib, the ROS1 inhibitor lorlatinib and the ROS1/NTRK inhibitor entrectinib have weak evidence suggesting clinical efficacy. Dabrafenib and vemurafenib both have an elevated lipid solubility, vemurafenib also has a small surface area and a long half-life while dabrafenib has a low IC_50_. Aside from the low molecular weight and surface area that is common to all PARP inhibitors, olaparib, talazoparib, and pamiparib also have a longer half-life than veliparib that was shown to have no clinical efficacy in phase 2 trials. Veliparib also has an elevated IC_50_ in GBM-specific cell lines. Abemaciclib, the CDK4/6 inhibitor with the highest lipid solubility and lowest surface area is the only drug with clinical efficacy in this class. Cabozantinib has a higher lipid solubility, lower surface area and lower IC_50_ than other RTK inhibitors and is the only one that was shown to have clinical efficacy. Osimertinib irreversibly binds to EGFR, has a high lipid solubility, small surface area, long half-life, and elevated maximal brain (tumor and non-tumor) concentrations. MW, molecular weight; IC_50_, half-maximal inhibitory concentration; Cmax, maximal concentration; MTD, maximal tolerated dose, *in preclinical studies.

We then focused on intra-drug class variations to understand differential therapeutic vulnerabilities (Figure 3). Among CDK4/6 inhibitors, abemaciclib has the highest lipid solubility and lowest SA (3.8 vs. class average of 2.6, 75 vs. class average of 89 Å^2^, respectively), which could support its ability to attain a high non-tumor brain concentration (3.6 μmoL/L) and brain-to-plasma ratio *in vivo* as well as explain its clinical efficacy in phase 2 trials (23, 24). PARP inhibitors are a class of drugs with low lipid solubility (mean 1.8), MW (mean 333 g/mol), and SA (mean 73.4 Å^2^) that have the ability to concentrate in non-tumor brain regions *in vivo* (25–27). Olaparib was the only PARP inhibitor shown to accumulate in contrast-enhancing brain lesions (28). Furthermore, talazoparib, olaparib, and pamiparib have demonstrated favorable clinical efficacy in brain tumors compared to veliparib which has a shorter half-life (6.1 h) and high IC_50_ (44.9 μM) (29–32). However, despite these features’ influence on the drugs’ clinical efficacy, there remains no evidence of their accumulation in non-enhancing brain regions.

Among KIT inhibitors, cabozantinib, exhibits notable clinical anti-tumor efficacy against GBM, in contrast to imatinib, tandutinib, and dasatinib, which have lower mean lipid solubilities, higher mean SAs, and comparable IC_50_ (mean 3.9 vs. 5.4, mean 104 vs. 98 Å^2^ and mean 34 vs. 1.8 μmoL/L respectively) (33–42). The BRAF inhibitors dabrafenib and vemurafenib have demonstrated clinical efficacy in patients with gliomas (43, 44), despite being substrates for efflux pumps (45). This may be in part attributed to their high lipid solubilities (4.8 and 5, respectively; Supplementary Table S3). Interestingly, while dabrafenib has a higher SA than vemurafenib (210 vs. 100 Å^2^), its low IC_50_ seems to offset this “unfavorable” feature. Together, these findings support the value of considering the overlay of multiple biochemical features in assessing the clinical efficacy of targeted agents for patients with brain tumors.

Finally, we speculated that the pharmacodynamic properties of targeted therapies, such as the reversible binding to its target, represent additional features that may impact the drug’s clinical efficacy. As a representative example, osimertinib, which is an irreversible inhibitor with a moderate lipid solubility (3.7 vs. class average of 3.1), that achieves high *in vivo* brain tumor (5.79 μmoL/L) and non-tumor brain concentrations (7.13 μmoL/L), has been shown to be clinically efficacious in case studies (46), despite the presence of efflux pumps (47). The significance of binding reversibility is also highlighted by the lack of clinical efficacy of the reversible EGFR inhibitors gefitinib, erlotinib, and lapatinib in phase 1–2 trials of patients with glioblastoma (48–53), despite sharing similar biochemical features with osimertinib. Overall, our findings point to the importance of considering multiple biochemical features of targeted therapies in clinical decision-making for patients with primary brain tumors.

### 3.3 Value of precision oncology in treating patients with primary brain tumors

To evaluate the clinical utility of molecularly-directed therapies for patients with high-grade gliomas and interpret potential therapeutic responses based on a nuanced genomic characterization in conjunction with the biochemical features of the targeted agent administered, we studied a cohort of 50 patients with high-grade gliomas reviewed at the Johns Hopkins Molecular Tumor Board (Methods). All primary brain tumors harbored at least one oncogenic mutation and consistent with the genomic profiles observed in TCGA cohort, *TP53* (52%), *NF1* (34%), *ATRX* (32%), *IDH1* (24%), *PIK3CA* (14%), *EGFR* (20%), and *BRAF* (8%) were frequently mutated. *EGFR, CDK4*, and *KIT* amplifications were found in 24, 16 and 10% of tumors, respectively, and *CDKN2A/2B* homozygous deletions were identified in tumors from 8 individuals (16%). Out of 540 alterations identified by NGS, 66 were structural alterations 474 were sequence alterations and of those 137 (29%) were classified as putatively oncogenic based on our approach (Methods). Among the 137 oncogenic sequence alterations, 63 (46%) were missense mutations and 74 (54%) represented loss of function mutations from nonsense, frameshift, and splice donor/acceptor site alterations (Supplementary Table S5). Out of 200 sequence and structural oncogenic alterations identified, 55.5% (*n* = 111) were characterized as targetable.

**Figure 4:**
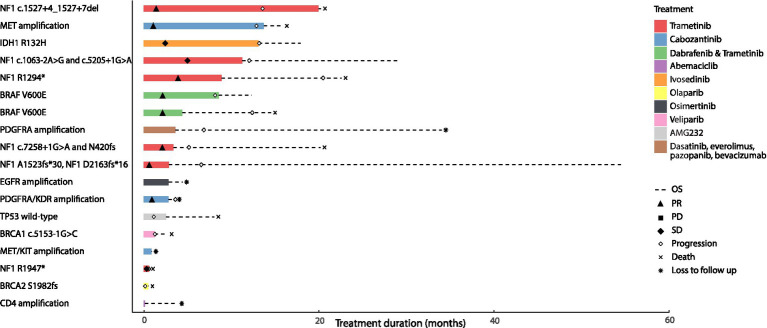
Clinical outcomes of patients with primary brain tumors harboring targetable alterations reviewed at the Johns Hopkins Molecular Tumor Board. This figure illustrates the survival outcomes (PFS and OS) of 18 patients with high-grade gliomas, treated with targeted agents after review and following targeted therapy recommendations at the Johns Hopkins MTB. The solid bar plot illustrates the duration of treatment in months, while bar colors correspond to the targeted drug administered. Overall survival is presented by a dotted line; death is illustrated with an X, and loss to follow up is presented with an * at the end of the survival line. Two patients had an *IDH1* mutant tumor; one was treated with ivosedinib and the other with cabozantanib for a *MET/KIT* amplification. Among the patients with *IDH* wild-type tumors, eight patients had radiographic partial response to the targeted therapy they received; four of them had received trametinib targeting an *NF1* alteration, two had received trametinib and dabrafenib targeting a *BRAF* V600E alteration and the last two had received cabozantinib targeting a *MET* amplification in one tumor and a *PDGFRA/KDR* amplification in the other. Two patients with tumors harboring an *NF1* alteration had radiographic stable disease on trametinib. Four patients with *PDGFRA* amplification, wild-type *TP53*, *BRCA1*, and *BRCA2* alterations had progressive disease as best radiographic response to targeted therapy and two patients with *EGFR* and *CDK4* amplification were lost to follow up before radiographic response could be assessed.

In this real-world cohort, among the 18 patients who received targeted therapy, there were two patients with *IDH1*-mutant high-grade astrocytoma that were excluded from our analysis based on the WHO 2021 classification of glioblastoma (54). Four patients with *NF1*-mutant high-grade glioma arising in the context of neurofibromatosis type 1 (*NF1* c.7258 + 1G > A and p.N420fs; *NF1* p.A1523fs*30 and *NF1* p.D2163fs*16; *NF1* c.1527 + 4_1,527 + 7del; *NF1* c.1063-2A > G and *NF1* c.5205 + 1G > A) and two patients with sporadic *NF1*-mutant high-grade glioma received trametinib monotherapy (*NF1* p.R1294*, *NF1* p.R1947*). Radiographic partial response (PR) or sustained stable disease (SD) was observed in 5 patients treated with trametinib monotherapy among which, 4 had neurofibromatosis type 1; one patient with *NF1* mutant sporadic glioma had SD followed by disease progression (Supplementary Table S4). Radiographic partial response was also noted in two patients with *BRAF* V600E-mutant high-grade glioma who were treated with dabrafenib and trametinib. Two patients with *MET* amplified and *PDGFRA/KDR* amplified glioblastoma were treated with cabozantinib and attained partial response. In contrast, four patients had progressive disease (PD); two patients with glioblastoma harboring *BRCA1* c.5153-1G > C and *BRCA2* p.S1982fs received a PARP inhibitor (veliparib and olaparib) and had PD after 1.4 and 0.2 months, respectively. One patient with glioblastoma had PD after 1.27 months while on AMG232 for wild-type *TP53* and another patient with glioblastoma harboring a *PDGFRA* amplification had PD after 7.53 months on dasatinib, everolimus, pazopanib and bevacizumab. The median overall survival in all 16 patients with *IDH1* wild-type high-grade gliomas that harbored actionable mutations and received genotype-matched targeted therapies, was 14.72 months with a median progression-free survival of 7.37 months (Figure 4).

Of the 32 remainder individuals, 3 were lost to follow up and one patient had a nonsense mutation in *PIK3R1* that while oncogenic, was not deemed targetable. Eleven patients with high-grade gliomas harbored an oncogenic *IDH1* mutation and were subsequently excluded from downstream analyses. Ten patients had tumors with multiple targetable alterations as follows: 8 tumors had a targetable *EGFR* alteration, 5 tumors had a *CDK4* amplification, 2 had an *MDM2* amplification and 1 had a *KIT/KDR/PDGFRA* amplification (Supplementary Table S4). Two tumors had an oncogenic *NF1* alteration and one had both an *NF1* and *ARAF* alteration. One tumor harbored a *BRAF* V600E alteration. Three tumors had an *FGFR* alteration; an oncogenic *FGFR1* sequence alteration, a *FGFR3* amplification and a *FGFR3-TACC3* fusion. Six tumors harbored alterations in the PI3K/AKT/mTOR pathway; four had a *PTEN* loss-of-function mutation and two had oncogenic *PIK3CA* alterations. One tumor harbored a *CDKN2A* homozygous deletion and one tumor had an oncogenic *BRCA2* alteration. With respect to therapeutic approaches; the majority of the patients received TMZ-based regimens (Supplementary Table S4). Of the 17 patients with available follow up, partial response was achieved in 12% (*n* = 2), stable disease was achieved in 53% (*n* = 9) and finally progressive disease was observed in 35% (*n* = 6; Supplementary Table S4). The median OS and PFS among patients with *IDH1* wild-type tumors harboring actionable mutations, who did not receive targeted therapy were 4.2 and 2.83 months, respectively. Taken together, our real-world analyses of patients with high-grade gliomas showed that patients matched to genotype-tailored therapies had better clinical outcomes compared to the ones that harbored putatively actionable alterations that did not receive genotype-targeted therapies.

## 4 Discussion

Despite a high frequency of sequence and structural alterations previously identified in GBM and the abundance of clinical trials investigating targeted therapies for patients with high-grade gliomas, the clinical efficacy of targeted agents has been largely disappointing to date (55). This phenomenon is multifactorial and in part driven by poor drug-target engagement, tumor heterogeneity, and inadequate accumulation of targeted agents in non-enhancing brain tumor tissue (56). To this end, understanding the host, tumor and drug features that affect targeted therapy efficacy is of paramount importance to drive the development of new treatment opportunities (57). In this study, we re-analyzed whole exome sequencing data from TCGA, focusing on the clonal heterogeneity of glioblastomas. In assessing the intra-tumoral heterogeneity of GBM, we found a higher proportion of recurring subclonal mutations compared to other tumor types; which may contribute to the suboptimal therapeutic response of GBMs to genotype-targeted therapies. Additionally, we leveraged these re-analyses focusing on the targetable genomic alterations captured and generated a comprehensive registry of annotated targeted therapies illustrating the complex interplay of their biochemical features in relation to their putative clinical efficacy.

Several studies have evaluated solitary features of drugs that affect the retention, the distribution, maximal intracranial concentration of drugs into the brain and consequently their efficacy in primary brain tumors, such as the presence of efflux transporters at the blood brain barrier, drug’s lipophilicity and molecular weight (58, 59), along with the affinity of the drugs to those transporters (60, 61). Our study highlights intra-class variations in the biochemical features, which could favor some targeted agents from others against the same genomic alteration. Importantly, when comparing the biochemical properties of temozolomide to the targeted agents evaluated in this study, temozolomide has the lowest MW (194.15), a poor lipid solubility (−1.1) and a moderate surface area (102 Å^2^). This observation further highlights the need to investigate and understand with more granularity, the properties that limit efficacy of targeted therapies in the brain. Notably, drug efficacy and activity should be assessed in molecularly driven preclinical models as differential growth inhibition can be observed in genomically characterized cell lines (62, 63).

One important feature to address in investigating the efficacy of targeted therapy in primary brain tumors, is the availability of the bound and unbound drugs in non-enhancing brain regions. Taking ribociclib as an example, the clinical accumulation of the free form of the targeted agent in non-contrast enhancing regions of the brain reaches close to 30% of its concentration in contrast enhancing regions (64). Our study highlights the significant knowledge gap in understanding the required optimal concentration that targeted agents should reach in tumor brain regions as well as in non-tumor brain regions to demonstrate clinical efficacy (4). There is therefore a need for additional Phase 0 studies to focus on identifying the targeted drugs’ ability to concentrate in non-enhancing brain regions prior to phase 2 studies and understand this drug feature as a potential surrogate of clinical efficacy (56). Additionally, the variabilities of target distribution may also impact mean residence time and drug accumulation (65).

In investigating the clinical efficacy of targeted therapies in a real-world based cohort of 50 patients with high-grade gliomas, we identified a significant rate of oncogenic targetable alterations detected by targeted NGS and highlight the pivotal role of NGS in identifying treatment vulnerabilities (66). Overall, the frequency of hotspot mutations in glioblastoma is comparable to that of tumors historically sensitive to molecularly directed therapies targeting these alterations. We identify trametinib alone or in combination with dabrafenib, as well as cabozantinib as successful drugs in the clinical setting against primary high-grade glioma with putatively targetable alterations as has been confirmed in phase 2 clinical trials (41–43, 67). While we hypothesize that the interplay between a prolonged half-life of these drugs as well as their high lipophilicity could favor the effective clinical efficacy, their contribution is not consistent. Previous studies have established that for a lipid-mediated free diffusion across the BBB, a MW threshold of 400 g/mol allows effective drug trafficking (59). However, a limited number of drugs evaluated in this study fit the dual criteria of a low MW and high lipid solubility. Therefore, the lack of a consistent implication of lipid solubility observed in our study could be explained by the large MW of those drugs. In addition, cabozantinib’s anti-VEGF activity could also explain the radiographic responses noted in our cohort (68).

Our study has several limitations; including restricting our analyses to genes mutated in >1% of primary brain tumors, which may have resulted in missing ultra-rare targetable alterations. In addition, recent discoveries have identified a self-renewal population of glioma stem cells that has high repopulating abilities and reflects the parent tumor heterogeneity, which are not accounted for in this study (69, 70). Given the paucity of data, we did not evaluate the ability of most targeted agents to accumulate in non-enhancing brain regions. Similarly, drug-tissue binding affinity which can also influence regional differences in free drug exposure in the brain tumor was also not investigated in this study (70). Furthermore, we acknowledge the limitations of the radiographic response assessment in our real-world cohort that did not use RECIST criteria rather relied on local radiology review as per standard of care.

Taken together, our findings support the notion that multiple host, tumor and drug-related features may limit the delivery and efficacy of targeted therapies directed to putatively actionable alterations in high-grade gliomas. Importantly, the paucity of reliable data on the ability of targeted agents to concentrate in non-enhancing tumor regions limits our understanding of their potential clinical efficacy in primary brain tumors. Future efforts are needed to investigate in a standardized manner the contribution of targeted agents’ biochemical features across all drugs and inform genomically-driven clinical trials for patients with primary brain tumors.

## Data availability statement

The datasets presented in this article are not readily available because of ethical restrictions to protect patient privacy. Requests to access the datasets should be directed to the corresponding author.

## Ethics statement

The studies involving humans were approved by Johns Hopkins Institutional Review Board. The studies were conducted in accordance with the local legislation and institutional requirements. Written informed consent for participation was not required from the participants or the participants’ legal guardians/next of kin in accordance with the national legislation and institutional requirements.

## Author contributions

PG: Conceptualization, Data curation, Formal analysis, Investigation, Methodology, Visualization, Writing – original draft. MF: Conceptualization, Data curation, Formal analysis, Investigation, Methodology, Visualization, Writing – original draft. DK: Conceptualization, Data curation, Formal analysis, Investigation, Methodology, Visualization, Writing – original draft. AB: Conceptualization, Data curation, Formal analysis, Investigation, Methodology, Visualization, Writing – original draft. MC: Data curation, Writing – original draft. JT: Supervision, Writing – original draft. JB: Supervision, Writing – original draft. JC: Investigation, Writing – original draft. SG: Conceptualization, Investigation, Methodology, Supervision, Writing – original draft. KM: Investigation, Writing – original draft. KS: Conceptualization, Data curation, Formal analysis, Investigation, Methodology, Supervision, Writing – original draft. VA: Conceptualization, Data curation, Formal analysis, Funding acquisition, Investigation, Methodology, Resources, Supervision, Visualization, Writing – original draft.

## The Johns Hopkins Molecular Tumor Board Investigators

, Christine Pratilas, Taxiarchis Botsis, Rena Xian, Chris Gocke, Ming-Tseh Lin, Eitan Halper-Stromberg, Ying Zou, Kent Hardart, Jonathan Spiker, Kory Kreimeyer, Ting He, Katie Fiallos, Dana Petry, Kala Visvanathan, Antonio Wolff, Cesar Santa-Maria, Raquel Nunez, Christian Meyer, John Laterra, Vered Stearns, Karen Smith, Deborah Armstrong, Rachel Karchin, Katerina Karaindrou, Lily Zandi, Marta Majcherska, Faith Too, Monique Makell, Jennifer Lehman, Timsy Wanchoo, Jaime Wehr, Michael Conroy and Selina Shiqing Teh.

## Footnotes

^1^ https://gdc.cancer.gov/about-data/publications/mc3-2017

^2^ https://gdc.cancer.gov/about-data/publications/pancanatlas
