# Supplementary material for: Druggable genomic landscapes of high-grade gliomas
Source: Front Med (Lausanne). 2023 Dec 8;10:1254955. doi: 10.3389/fmed.2023.1254955 (PMC10749203; doi:10.3389/fmed.2023.1254955)
Supplement: Supplementary file 1 [file Data_Sheet_1.docx]

**Supplementary Figures**

**Supplementary Figure S1. Flow diagram illustrating the overall approach of the study.** The upper portion of the flow diagram summarizes the analyses performed on the TCGA dataset of 388 glioblastomas. The middle portion illustrates the characteristics extracted for 54 targeted therapies. Biochemical features were collected from PubChem, while *IC_50_ was extracted from selleckchem.com. Evidence on the presence of efflux pumps at the blood brain barrier along with the brain-to-blood ratio were extracted by performing a literature search. The drugs’ concentration in the brain (tumor, non-tumor and CSF) as well as in the plasma of *in vivo* models and in humans were collected from published data. The lower portion of the diagram summarizes the methodology applied to the real-world cohort of patients with brain tumors, pertaining to the variants’ annotation and the survival data of patients who have received targeted therapies.

**Supplementary Figure S2. Actionable genomic landscape of glioblastoma.** This landscape figure illustrates the nonsynonymous mutations of glioblastomas from 388 tumors in the TCGA dataset. A breakdown by type (missense, nonsense, indels) as well as amplifications and homozygous deletions are illustrated for each gene, ordered by 11 signaling pathways/cancer hallmarks. Glioblastomas did not harbor concurrent hotspot mutations and copy number alterations in each respective gene with the exception of 2.4% of *EGFR*-mutant tumors that harbored concurrent sequence alterations and copy number gains in *EGFR*. The bar plots on the right summarize the frequency of mutation types per gene and the percentage on the top of each bar plot, represents the proportion of tumors harboring 1 or more mutation in each respective gene. The most commonly altered genes across all tumors included *CDKN2A* (52.5%), *CDKN2B* (50.5%), *PTEN* (41.1%), *EGFR* (28.3%), *TP53* (31.6%), *NF1* (12.5%) and *PIK3CA* (9.5%), followed by less frequent sequence alterations in *CDK4* (7.1%), *IDH1* (6.8%), *PDGFRA* (5.4%), *BRAF* (2.2%), *BRCA2* (1.6%), and *EZH2* (1.1%). Copy number gains were observed in *CDK4* (7.1%), *EGFR* (7.1%), *MDM4* (4.3%), *KIT* (2.7%), *KDR* (1.6%) and *PDGFRA* (1.6%), while the most frequently identified homozygous deletions were observed in *CDKN2A* (51.5%), *CDKN2B* (49.9%), *PTEN* (9.0%), and *NF1* (1.1%). Sub-clonal mutations are tagged with a cross. Hotspot mutations are also illustrated in the genomic landscape and are tagged with a square.

**Supplementary Figure S3. Evaluation of the fraction of recurring (hotspot) mutations relative to their clonality across multiple cancer types from TCGA.** This figure illustrates the comparable fraction of hotspot mutations across several cancer types including GBM (29.9%). Hotspot mutations were defined by an entry of ≥10 in COSMIC. An important fraction of hotspot mutations is found in pancreatic adenocarcinoma (46.7%), breast cancer (40.2%), head and neck cancer (37.9%), ovarian cancer (38.9%), colon cancer (30.9%), lung adenocarcinoma (28.2%), bladder cancer (24.0%) and melanoma (24%). This figure also highlights comparable rates of sub-clonal mutations in TCGA. Importantly, lower rates of sub-clonal mutations were observed in pancreatic cancer (11.6%), melanoma (11.6%) breast cancer (9.4%), colon cancer (8.4%), bladder cancer (8.2%), lung cancer (6.1%) and head and neck cancer (5.6%) compared to GBM (15.3%). Abbreviations: UCEC, Uterine Corpus Endometrial Carcinoma; SKCM, Skin cutaneous melanoma; COAD, colon adenocarcinoma; STAD, Stomach adenocarcinoma; BLCA, urothelial bladder carcinoma; LUAD, lung adenocarcinoma; LUSC, lung squamous cell carcinoma; BRCA, breast invasive carcinoma; LGG, low-grade glioma; HNSC, head and neck squamous cell carcinoma; GBM, glioblastoma; OV, ovarian cancer; READ, rectal adenocarcinoma; LIHC, liver hepatocellular carcinoma; ESCA, esophageal carcinoma; PAAD, pancreatic adenocarcinoma; KIRC,  Kidney Renal Clear Cell Carcinoma; THCA, thyroid cancer; KIRP, Kidney renal papillary cell carcinoma; SARC, sarcoma; UCS, uterine carcinosarcoma; prostate adenocarcinoma; ACC, Adrenocortical carcinoma; MESO, mesothelioma; CHOL, Cholangiocarcinoma; DLBC, diffuse large B cell lymphoma; KICH, Kidney Chromophobe; TGCT, testicular germ cell tumors; PCPG, Pheochromocytoma and paraganglioma; UVM, uveal melanoma; THYM, thymoma

**Supplementary Figure S4. Heatmap of all biochemical features considered for 54 genotype-targeted therapies.** This figure illustrates the different biochemical features of 54 targeted therapies against the most commonly occurring genomic alterations (≥1%) in glioblastoma. In addition to the previously mentioned features in Figure 3, this heatmap includes the brain and blood distribution of targeted therapies from clinical studies among which the maximal plasma concentration, the maximal drug concentration in the brain enhancing and non-enhancing tumors as well normal brain tissue, the maximal CSF concentration and CSF to plasma ratio. MW, molecular weight; IC_50_, half-maximal inhibitory concentration; Cmax, maximal concentration; CSF, cerebrospinal fluid; MTD, maximal tolerated dose; *in preclinical studies; #in clinical studies
